# Analysis of Compressive Fatigue Failure of Recycled Aggregate Concrete

**DOI:** 10.3390/ma14164620

**Published:** 2021-08-17

**Authors:** Fan You, Surong Luo, Jianlan Zheng, Kaibin Lin

**Affiliations:** 1 College of Civil Engineering, Fuzhou University, Fuzhou 350116, China; 2015032@fjjxu.edu.cn (F.Y.); jianlan@fzu.edu.cn (J.Z.); 2 College of Engineering, Fujian Jiangxia University, Fuzhou 350108, China; 2016012@fjjxu.edu.cn

**Keywords:** recycled aggregate concrete (RAC), fatigue life, Weibull distribution, fatigue deformation, nanoindentation, interfacial transition zone (ITZ)

## Abstract

Using recycled aggregate in concrete is effective in recycling construction and demolition waste. It is of critical significance to understand the fatigue properties of recycled aggregate concrete (RAC) to implement it safely in structures subjected to repeated or fatigue load. In this study, a series of fatigue tests was performed to investigate the compressive fatigue behavior of RAC. The performance of interfacial transition zones (ITZs) was analyzed by nanoindentation. Moreover, the influence of ITZs on the fatigue life of RAC was discussed. The results showed that the fatigue life of RAC obeyed the Weibull distribution, and the *S-N-p* equation could be obtained based on the fitting of Weibull parameters. In the high cycle fatigue zone ($N\geq 10^{4}$), the fatigue life of RAC was lower than that of natural aggregate concrete (NAC) under the same stress level. The fatigue deformation of RAC presented a three-stage deformation regularity, and the maximum deformation at the point of fatigue failure closely matched the monotonic stress-strain envelope. The multiple ITZs matched the weak areas of RAC, and the negative effect of ITZs on the fatigue life of RAC in the high cycle fatigue zone was found to be greater than that of NAC.

## 1. Introduction

Concrete is one of the most widely used building materials. The annual world concrete production varies depending on the source between 13 billion and 21 billion tons [1]. Mass production of concrete leads to excessive consumption of natural resources, especially natural aggregates, which account for about 80% of the total volume of concrete [2]. At the same time, the construction and demolition of structures lead to excessive waste. The total global annual production of construction and demolition waste (CDW) has been reported to exceed 3 billion tons [3]. How to deal with this CDW has become a global problem. At present, the prime disposal methods of CDW are stacking and landfill, which not only occupy land space, but also cause serious pollution to the environment. In terms of ecological protection and effective utilization of resources, the recycling of CDW is unquestionably valuable [4]. As waste concrete accounts for the largest proportion of CDW, crushing and grading waste concrete into recycled concrete aggregate (RCA) is an effective and important method to achieve CDW recycling and sustainable development within the construction industry. This is accomplished by partly or totally replacing natural aggregate with RCA in the preparation of recycled aggregate concrete (RAC) [5,6,7].

As is widely acknowledged, natural aggregate concrete(NAC) is composed of hardened cement paste, aggregate, and an interfacial transition zone (ITZ) between the aggregate and cement paste. ITZ is the weak link within concrete due to its high porosity and numerous micro cracks, which significantly impacts on the performance of concrete materials [8]. RCA usually consists of primary aggregate, adherent old mortar, and ITZs between the adherent old mortar and primary aggregate [9,10]. The old mortar attached to the surface of RCA determines the complexity of interface conditions in RAC. For example, there are ITZs between RCA and old cement mortar, between RCA and new cement mortar, and between old and new cement mortar [11,12,13]. The existence of multiple ITZs is the main reason for the differences in mechanical properties between RAC and NAC [8,14].

Some concrete structures such as bridges, highway pavements, and dams are often required to bear cyclic loading in addition to static loading, and may suffer brittle failure before reaching their static load strength, namely fatigue failure [15,16,17,18,19]. Fatigue failure is one of the most common failure modes of concrete structures. In order to ensure the reliability and safety of RAC in practical engineering applications under cyclic loading, it is essential to study its fatigue performance. Xiao et al. [20] investigated the fatigue performance of RAC under uniaxial compression with 100% replacement with recycled coarse aggregate, focusing on the strain response and fatigue damage accumulation under fatigue load. They concluded that under equal stress levels, the fatigue life of RAC lasted longer than that of NAC. Changes in fatigue strain were found to reflect the damage to the recycled concrete, and the fatigue modulus was used to describe the progression of damage to the RAC.Thomas et al. [21,22] discussed the fatigue properties of RAC containing 0%, 20%, 50% and 100% of recycled coarse aggregate. They found that recycled coarse aggregate had a more negative effect on the fatigue performance of concrete than on its static strength, and that recycled aggregate could reduce the fatigue life of concrete under comparable water to cement ratio. These findings were contrary to the conclusions of Xiao et al. [20]. This was mainly because fatigue damage is essentially a random dynamic process dependent on the time variables and randomness of the static load strength of concrete materials, the unpredictability of initial defects, of defect propagation, and of damage and fracturing. The fatigue life of concrete is also highly discrete and may vary by several orders of magnitude, for instance from 100 to 100,000 cycles [23]. Moreover, due to the diversity of sources of recycled coarse aggregate, the complexity of service conditions of waste concrete, and the multiplicity of ITZs in RAC, the performance of RAC is more variable than that of NAC [24,25,26,27]. However, in the existing studies on the fatigue performance of RAC under uniaxial compression, the test sample base was relatively small and thus failed to consider the statistical properties of concrete fatigue life [23,28,29,30,31,32,33]. The theory of probability was furthermore not used to estimate and analyze the fatigue life of RAC, which might have reduced the impact of such discreteness on the test results.

In order to provide an applicable research basis to RAC in structures under fatigue load in this study, a fatigue test of RAC with 100% replacement with recycled coarse aggregate under uniaxial compression load was performed, and the Weibull distribution of fatigue life under different stress levels was verified. The fatigue life equations under different survival rates were established, and the basic deformation regularity was scrutinized. Finally, through nanoindentation analysis, the performance of ITZs in RAC and their influence on fatigue life were discussed.

## 2. Experimental Design

### 2.1. Specimen Preparation

The recycled coarse aggregates were made from crushed waste concrete from former pavement structures. Particle sizes ranged from 5.0 to 20.0 mm, and the apparent density was 2620 kg/m${}^{3}$. In addition, the crushing index was 10.05, and the water absorption was 5.1%. The fine aggregate was natural river sand with a fineness modulus of 1.90. The cement was P·O 42.5 ordinary Portland cement with a density of 3150 kg/m${}^{3}$ and a specific surface area of 352 m${}^{2}$/kg. The type of fly ash was Grade II with a fineness of 13% (45 μm of sieve residue) and a water requirement of 95%. The water reducer was polycarboxylate superplasticizer, and the water was ordinary tap water.

The mix proportion of RAC is shown in Table 1. The coarse aggregate was 100% composed of RCA. The volume of water was increased in consideration of the high water absorption of recycled coarse aggregate. The ratio of sand to coarse aggregate was 0.38, and the actual effective water to binder ratio was 0.40. Cubic and prism specimens sizes used for uniaxial compression tests were 150 mm × 150 mm × 150 mm and 100 mm × 100 mm × 300 mm, respectively. The size of prism specimens used for fatigue tests was also 100 mm × 100 mm × 300 mm. These specimens were demolded after 24 h’casting and cured in a standard curing room for 28 days, after which they were placed in a dry indoor environment for 90 days to eliminate the influence of age on the fatigue properties of materials.

### 2.2. Test Method

**Figure 1:**
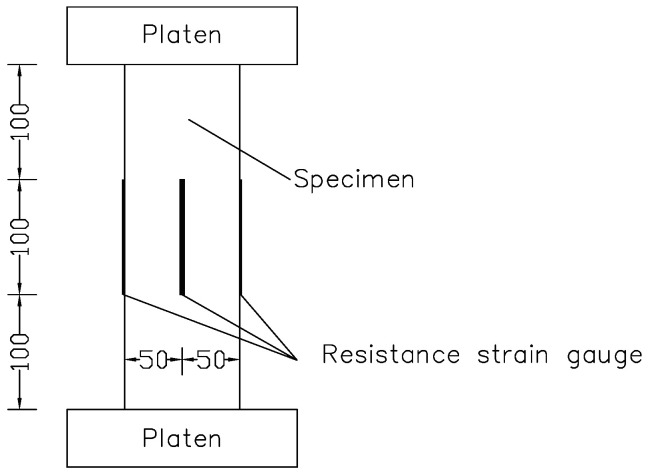
Setup of fatigue test(mm).

The displacement-control loading mode was adopted for the static load testing of prism specimens under uniaxial compression, and the loading rate was 0.2 mm/min. Fatigue testing was carried out in accordance with the 1000 kN high performance dynamic fatigue testing system produced by the company MTS, and the load-control loading mode was adopted. A sinusoidal reciprocating cyclic constant amplitude load was applied with a loading frequency of 4 Hz. The setup of fatigue test was shown in Figure 1. As can be seen from Figure 1, a 100 mm long resistance strain gauge was uniformly installed at the middle height of the sample to measure the axial strain at the middle position of the sample. The collected test data included the load of the testing machine throughout the entire process of static load and fatigue testing, the relative displacement of the upper and lower platens, and the strain of the strain gauge pasted at the center of the specimens. The sampling frequency of these test data was 50 Hz.

The uniaxial static load test was performed on RAC first, that is, before the fatigue test. The average cubic compressive strength was $f_{c}=55.7$ MPa (3 specimens), and the mean prism compressive strength was $f_{u}=50.3$ MPa (5 specimens), while the average elastic modulus measured in the static load test was 31.6 GPa (3 specimens). In order to study the fatigue characteristics of RAC materials under high and low fatigue cycles, four stress levels were considered in the test, which were 0.90, 0.85, 0.80, and 0.75, respectively. The stress level *S* in the fatigue test was the ratio of the maximum fatigue stress $\sigma_{max}$ to the mean static load compressive strength $f_{u}$ of the specimen. Therefore, the corresponding maximum fatigue stresses were 45.27 MPa, 42.76 MPa, 40.24 MPa, and 37.73 MPa, respectively. In addition, the minimum fatigue stress of fatigue loading was kept at 0.1 $f_{u}$. Fatigue tests were performed on 5 samples per stress level. At the beginning of the fatigue test, the rate tended toward the average load at a rate of 8 kN/s, following which the specimen was either damaged or continued to bear fatigue load up to 2 × 10${}^{6}$ cycles under cyclic load by sinusoidal fatigue loading. The test was stopped, and the fatigue life of the concrete was recorded by the instrument.

The indentation moduli of three ITZs of RAC were measured with the Hysitron Ti Premier nanoindentation test system. Before the test, all RAC samples were processed, including cutting, epoxy resin inlay, grinding, and polishing.

Firstly, a cutting machine was used to cut 15 mm × 15 mm × 15 mm small cubes, which were then soaked in ethanol for 24 h. After taking them out, they were heated at 50 ${}^{\circ }$C for 12 h.

Secondly, the samples were inlaid with epoxy resin. When the epoxy resin hardens, it stabilizes the microstructure of RAC so that it can withstand the stress of grinding and polishing without any change in the microstructure.

Finally, the inlaid samples were ground and polished using a full-automatic grinding and polishing machine to make the surface as flat as possible, so as to reduce the interference from samples. In the grinding process, P600 and P1200 sandpapers were used, and water was utilized as a cooling medium and lubricant. After polishing and cleaning treatment, the samples were polished with diamond oil suspensions of three different granularities (i.e., 9 μm, 3 μm, and 15 μm).

After the samples were fabricated, the optical microscope installed on the nanoindentation instrument was used to observe their surface. With higher magnification, the composition of different phases of RAC, including aggregate and new/old mortar, can be observed. The ITZs between aggregate and new mortar, aggregate and old mortar, and old and new mortars were called ITZ${}_{1}$, ITZ${}_{2}$, and ITZ${}_{3}$, respectively. The matrix dot was used for the nanoindentation test, and a 100 μm × 100 μm square matrix was used for ITZ${}_{1}$ and ITZ${}_{2}$. The distance between each indentation point pairs was 10 μm, i.e., the indentation was an 11 × 11 lattice. Furthermore, a 150 μm × 100 μm square matrix was used for ITZ${}_{3}$, and the distance between each indentation point pair was also 10 μm, with a 16 × 11 lattice.

The optical microscope (10×) installed on the nanoindentation instrument was used to find the view that contained ITZs. Afterwards, the starting point of the nanoindentation lattice and the corresponding indented area was located using the intersection of two mutually perpendicular blue coordinate lines.The force-controlled loading mode was adopted in the experiment. At a loading rate of 200 μN/s, the maximum load of 2000 μN was reached after 10 s. After keeping the maximum load for 5 s, the samples were unloaded to 0 at a rate of 200 μN/s.

## 3. Results and Analysis

### 3.1. Fatigue Life and Distribution

Fatigue life refers to the number of cycles required to induce fatigue failure to a material under cyclic loading. Table 2 shows the test results for RAC under uniaxial compression fatigue life at different stress levels. In previous studies, the Weibull distribution was usually used to study the fatigue life of concrete [23,28,33,34,35]. Thus in this study, the Weibull distribution was also applied to investigate the fatigue life of RAC. When the location parameter or the minimum life parameter was 0, the two-parameter Weibull cumulative distribution function $P_{f}$ could be written as follows:

(1) $$P_{f}=e^{-{\left(\frac{N}{N_{a}}\right)}^{b}}$$

where: $P_{f}$ is the failure probability; *N* is the fatigue life, $N_{a}$ is the characteristic parameter of fatigue life; and *b* is the shape parameter of Weibull distribution.

After taking twice natural logarithms of Equation (1), the following equation could be obtained:

(2) $$\ln\left[\ln\left(\frac{1}{1-P_{f}}\right)\right]=b\ln N-b\ln N_{a}$$

If $Y=\ln\{\ln\left[1/\left(1-P_{f}\right)\right]\}$, X=lnN, and $c=b\ln N_{a}$, then Equation (2) could be simplified as:

(3) Y=bX−c

**Figure 2:**
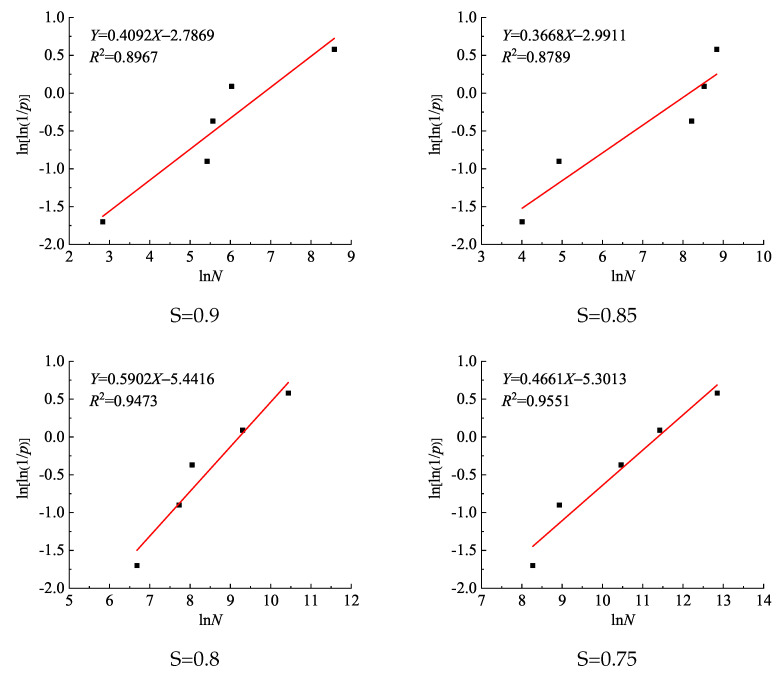
Relationship of ln[ln(1/*p*)] with ln*N* under each stress level.

Equation (3) could be used to test whether a group of fatigue life test data conformed to the two-parameter Weibull distribution. If using graphical regression analysis, the linear relationship between *Y* and *X* proved good, the hypothesis that fatigue life complies with the Weibull distribution was deemed tenable. According to the fatigue life shown in Table 2, the corresponding survival rate *p* could be calculated:

(4) $$p=1-P_{f}=\frac{i}{m+1},\left(i=1,2,\ldots \ldots ,m\right)$$

where: *i* is the number of fatigue life scale in ascending order from small to large under the same stress level; *m* is the sample size of the fatigue test at a given stress level. Then, the fatigue life at different stress levels was regressed linearly according to Equation (3). The regression analysis of the relationship of ln(ln(1/*p*)) with ln*N* under each stress level is shown in Figure 2. It can be seen from Figure 2 that at all levels of stress, the correlation coefficient *R${}^{2}$* was between 0.8789 and 0.9551, and the linear relationship between *X* and *Y* was significant, indicating that the compressive fatigue life of RAC materials was found to be subject to the Weibull distribution. Table 3 below lists the Weibull distribution parameters of RAC fatigue life at different stress levels. From shape parameter *b*, it can be seen that the influence of fatigue stress level on the dispersion of RAC fatigue life was not significant.

In addition, in the engineering application of concrete, it is generally necessary to establish the *S*-*N* equation under a specified survival rate according to the reliability requirements. According to Equation (2), the fatigue life of concrete could be expressed as:

(5) $$N=N_{a}{\left[\ln\left(\frac{1}{p}\right)\right]}^{1/b}$$

Through Equation (5), the corresponding fatigue life *N* of concrete under a certain stress level *S* could be calculated under a given survival rate *p*.

To ensure the reliability of the predicted life of concrete at low stress levels, the following single logarithm equation could be used to express the fatigue life:

(6) S=A+BlogN

where A and B are the equation parameters.

**Figure 3:**
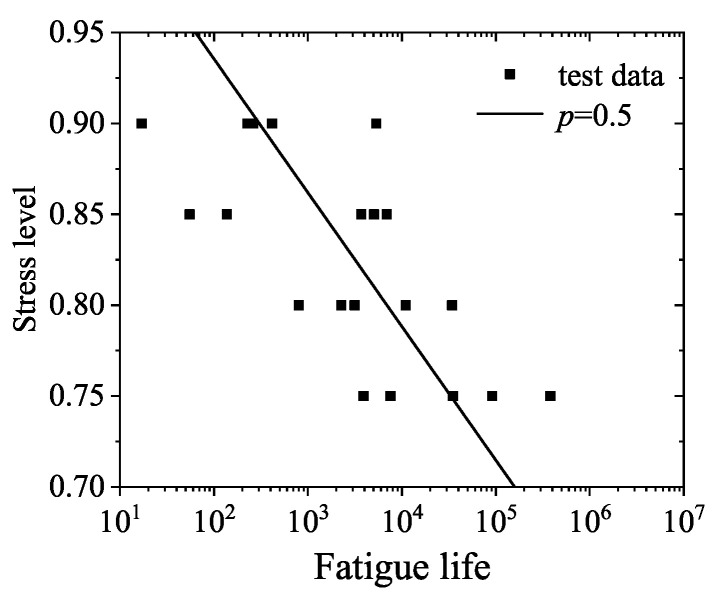
Experimental fatigue life and fatigue curve of RAC.

The relationship curve of *S*-*N*, that is, the *S*-*N*-*p* equation, can be obtained by linearly regressing the fatigue life under different failure probabilities in the form of Equation (5). The single logarithm fatigue equation of RAC under different survival rates *p* is shown in Table 4. In addition, the tested fatigue life and fatigue equation of RAC are plotted in Figure 3.

In order to compare RAC with natural aggregate concrete of similar compressive strength, a Weibull distribution analysis was performed on the uniaxial compression fatigue life test data of two groups of natural aggregate concrete (NAC-1 and NAC-2) in references [36,37,38] with cubic compressive strengths of 54.2 MPa and 46.8 MPa, respectively. According to these analyses, the *S-N-p* equations of NAC-1 and NAC-2 were obtained using the same process as stated above. The fatigue lives of RAC and NAC under a survival rate p=0.5 under each stress level calculated by Equation (5) are given in Table 5. The fatigue equation obtained by the fitting of a single logarithm equation is listed in Table 4. It can be seen from Table 4 that the correlation coefficients *R${}^{2}$* of the fatigue equations of NAC-1 and NAC-2 were greater than 0.99, with a high confidence level.The mix proportion of NAC-1 and NAC-2 are shown in Table 6 (The fine aggregate was made of natural river sand with a moisture content of 3.8 percent).

**Figure 4:**
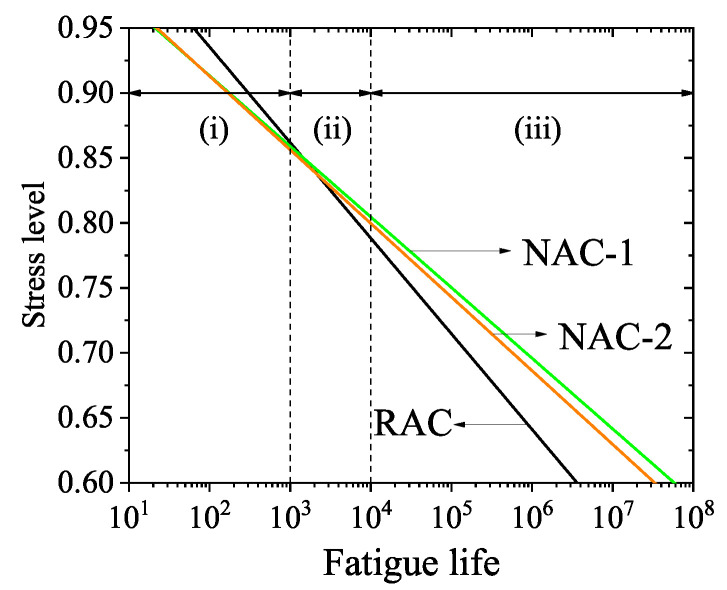
*S*-*N* curve of RAC and NAC under compression fatigue load.

The RAC and NAC fatigue equations under the survival rate *p* = 0.5 are shown in Figure 4. It can be seen from Figure 4 that under the same stress level, the fatigue life of RAC was found to be lower than that of the NAC-1 and NAC-2 in the high cycle fatigue zone, with $N\geq 10^{4}$, and higher than that of NAC-1 and NAC-2 in the low cycle fatigue zone, with $N\leq 10^{3}$.

Under the action of fatigue loading, the failure process of the concrete material essentially matched the process of crack evolution and development in the ITZ and matrix. According to the International Union of Laboratories and Experts in Construction Materials, Systems and Structures (RILEM) report, the fatigue failure of concrete materials is attributed to two mechanisms: the degradation of the bond between the matrix and coarse aggregate, and the development of cracks in the matrix. These two failure mechanisms either act alone or coexist in time [39]. Hsu [40] considered that for low cycle and high amplitude fatigues, the matrix failure was dominant, and the continuous “through-cracks” that had formed due to the fatigue crack extending into the mortar had led to the final failure. For high cycle and low amplitude fatigues, the debonding of the interface between matrix and aggregate had led to the failure of the material, and the slow and gradual development of bonding cracks between mortar and coarse aggregate had resulted in the material fatigue failure. In addition, Zheng [41] further subdivided the fatigue component into three zones by introducing the influence factors of matrix and ITZs into the fatigue performance. For the low cycle fatigue zone corresponding to $N\leq 10^{3}$, the dominant mechanism of fatigue damage was matrix cracking, and the matrix property was deemed the main factor affecting fatigue life, which was named the dominant matrix cracking zone. In the high fatigue zone cycle corresponding to $N\geq 10^{4}$, the bond cracking initiated, propagated to the interfacial zone, and extended to the matrix was deemed the dominating reason for fatigue failure. Thus, the properties of the interfacial zones were the main factors affecting fatigue life. These zones are called the dominant bond cracking zones. Correspondingly, in the region of $10^{3}<N<10^{4}$, the fatigue damage to concrete material mainly developed due to matrix cracking within the interfacial zones. The properties of the matrix and interfacial zone were both found to significantly impact on the fatigue performance of concrete, which region is called the transition zone. Accordingly, the fatigue zoning of concrete in Figure 4 was achieved as follows: (i) matrix cracking control zone, (ii) transition zone, and (iii) interface cracking control zone.

**Figure 5:**
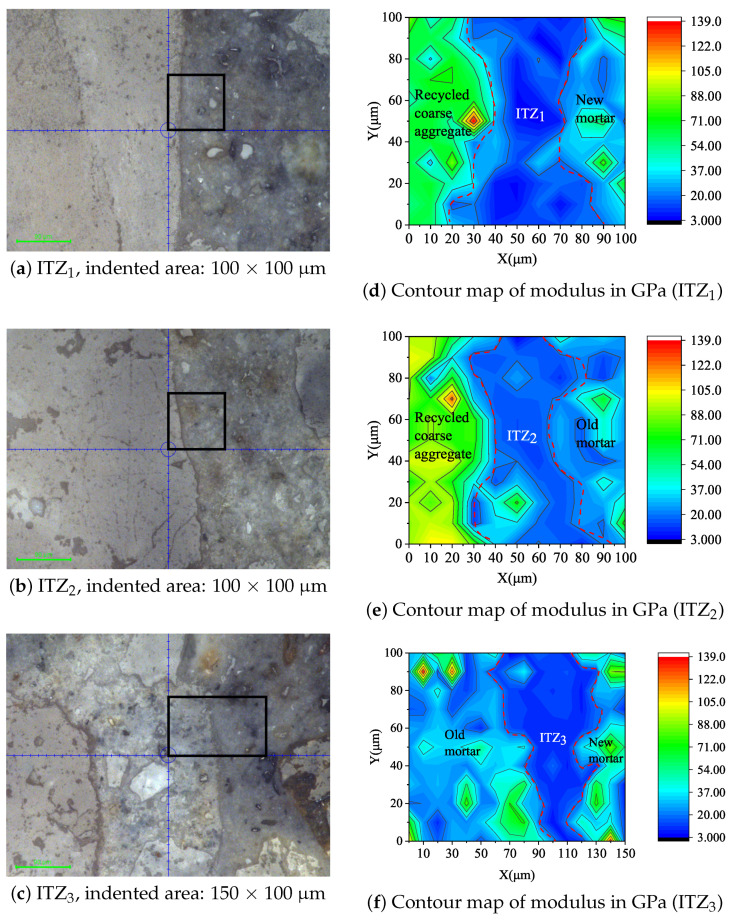
Grid indentation across ITZs in RAC at 90 days.

In the high fatigue cycle region ($N\geq 10^{4}$), the fatigue life of RAC was lower than that of the NAC-1 and NAC-2, which mainly related to the nature of the multiple ITZs of RAC. Nanoindentation tests were conducted on the ITZ of RAC at the age of 90 days. The selected indented areas on the sample are shown in Figure 5a–c, containing ITZ${}_{1}$, ITZ${}_{2}$ and ITZ${}_{3}$ of RAC, respectively. After the indentation test, the obtained modulus lattice data were processed using the function of Contour-Color fill in the Origin software. Thus, the contour maps of indentation modulus of different ITZs were obtained, as shown in Figure 5d–f. It can be seen from Figure 5 that there are three kinds of ITZs (ITZ${}_{1}$, ITZ${}_{2}$ and ITZ${}_{3}$) in RAC.In any ITZ, the indentation modulus is lower than that of the new and old mortars, which indicates that the three kinds of ITZs are weak connecting components of RAC. Due to the existence of multiple weak ITZs, RAC is more prone to produce bond cracks than NAC in the crack control zone of the interface zone, which has a more detrimental effect on fatigue life.

In the low fatigue cycle region ($N\leq 10^{3}$), the fatigue life of RAC was found to be higher than that of NAC-1 and NAC-2. The water to binder ratio is an important factor determining the porosity of concrete matrix, and the existence of pores has an adverse effect on the concrete matrix performance [42]. It can be seen from Table 1 and Table 2 that the effective water to binder ratio of RAC is 0.4, while that of NAC-1 and NAC-2 is much greater than 0.4 (the sand used for RAC is dried sand with a water content of about 0, while the mass water content of sand used for NAC is 3.8%). Thus, the effective water to binder ratio of RAC is lower than that of NAC-1 and NAC-2. This suggests that the RAC matrix has a lower porosity than NAC-1 and NAC-2, and its matrix property is better, having a stronger ability to resist crack development. Meanwhile in the crack control zone of matrix, the matrix property is the main factor affecting the fatigue life of concrete. This may be the main reason that the fatigue life of RAC is higher than that of NAC-1 and NAC-2 in the low cycle fatigue region ($N\leq 10^{3}$). Moreover, it is the main factor that the cube compressive strength of RAC is higher than that of NAC-1 and NAC-2.

In the matrix cracking control zone (i), the fatigue life of RAC is higher than that of NAC-1 and NAC-2. However, in the interface cracking control zone (iii), the fatigue life of RAC is lower than that of NAC-1 and NAC-2, and this gap increases gradually with the decreasing stress level. In another way, it suggests that in the high cycle fatigue region ($N\geq 10^{4}$), the existence of multiple ITZs has a greater adverse effect on the fatigue life of RAC than NAC.

### 3.2. Fatigue Failure Deformations

**Figure 6:**
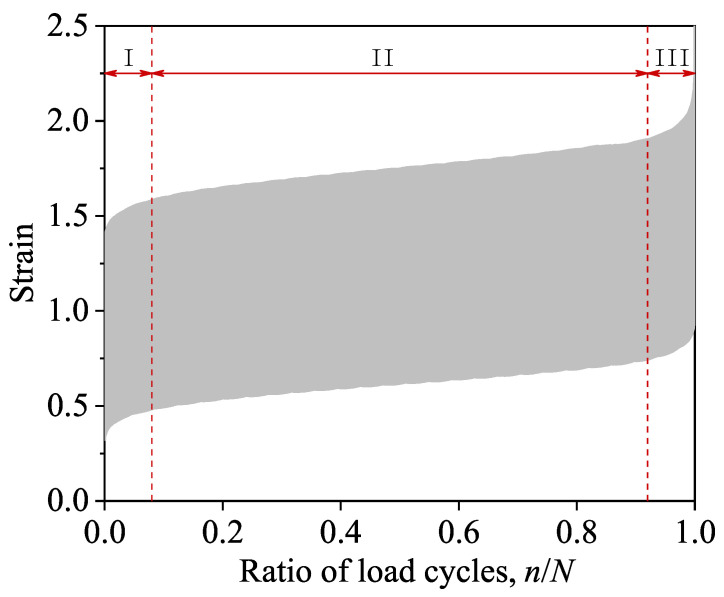
Evolution curves of deformation of fatigue specimen with ratio of load cycles.

Figure 6 shows the typical strain evolution curve of fatigue specimen F-0.85-4 (*n* is the number of cycles; *N* is the fatigue life of the specimen). It can be found from Figure 6 that the compressive fatigue deformation of the recycled aggregate concrete was similar to that of the natural aggregate concrete [43], showing a characteristic three-stage development regularity. Stage (I) was a rapid developing stage in which the strain increased rapidly from 0, and which stage accounted for about 10% of the total life. Stage (II) was a stabilizing development stage in which the strain increases slowly; this stage accounted for 80% of the total life. Stage (III) was the failure stage in which the strain increased rapidly, which stage accounted for about 10% of the total life. Similar phenomena were also found in all the remaining specimens with stress levels from 0.70 to 0.90.

**Figure 7:**
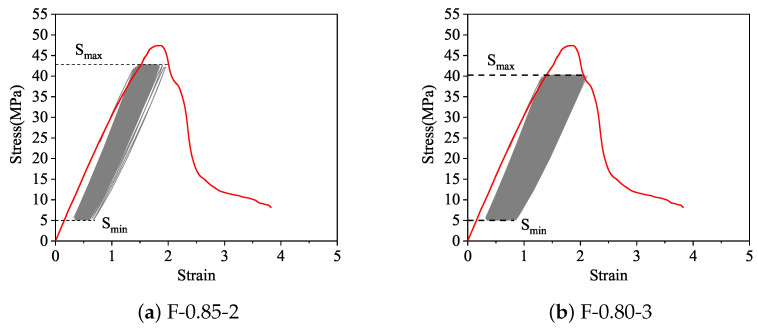
Comparison of maximum strain at fatigue failure of RAC with monotonic envelope.

Figure 7 shows the stress-strain curves of fatigue specimens F-0.85-2, F-0.80-3 and monotonic static load specimens. It can be seen from Figure 6 that the general characteristics of the cyclic stress-strain curve of recycled aggregate concrete are similar to that of the natural aggregate concrete [43]. With the increase of the cycle number, the stress-strain curve “flows” to the direction of increasing deformation, and shows the obvious three-stage development characteristics of sparse-dense-sparse. At the same time, the closer to the fatigue damage limit state, the greater the inclination of the stress-strain curve, which obviously protrudes to the deformation axis.

The maximum deformation at fatigue failure is generally defined as the deformation corresponding to the maximum load of the last fatigue cycle before the specimen fails. Available studies have shown that the fatigue deformation of natural aggregate concrete is characterized by the deformation envelope [43]. In addition, the envelope coincides with the monotonic load curve or at least closely approximates it. It is clear from Figure 7 that the maximum deformation of RAC at failure is very consistent with the monotonic envelope. Although not shown in the figure, the same conclusion was obtained in respect of other stress levels. Therefore, the monotonic stress-strain curve of RAC can be used as the fatigue deformation in respect of the envelope.

### 3.3. Fatigue Failure Characteristics

**Figure 8:**
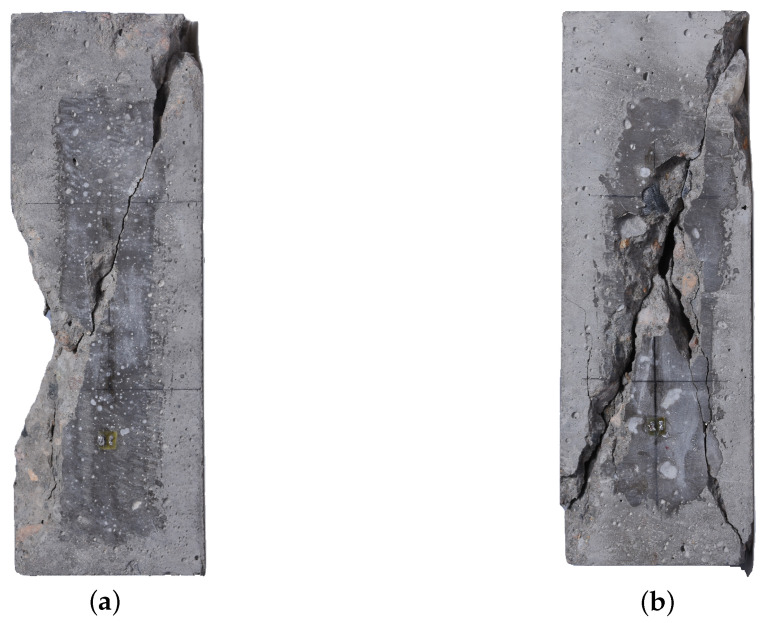
Typical fatigue failure modes of RAC under compression. (**a**) F-0.8-3; (**b**) F-0.75-1.

Figure 8 shows the typical fatigue failure mode of RAC specimens under uniaxial load. It can be seen that the main inclined shear crack formed along the specimen inclined to about 60${}^{\circ }$ relative to the horizontal plane. As with natural aggregate concrete [44], the fragmentation in the middle of the specimen was bursting in all directions at the moment of RAC fatigue failure, revealing evidence of brittle failure characteristics.

**Figure 9:**
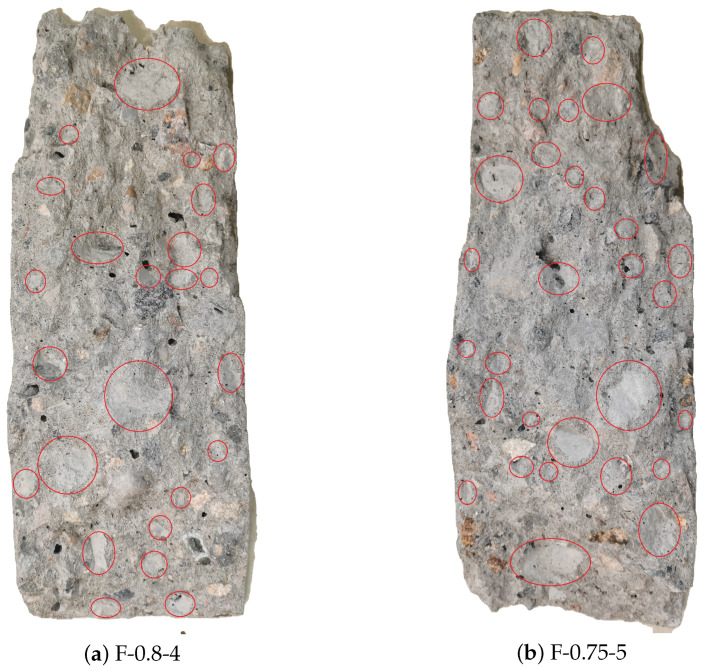
Fatigue fracture surface of RAC.

Figure 9 below shows the fracture surface of RAC specimens (F-0.8-4, F-0.75-5) under fatigue failure. It can be seen that the aggregate on the fracture surface shows several debonding failure characteristics. In addition, the lower the stress level and the higher the number of cycles, the more evident the debonding failure characteristics appear. The main reason for this is that under the action of low stress levels, cracks develop along the interface between mortar and aggregate, and concrete fatigue cracks have enough cycles to develop into the ITZ, inducing numerous large bonding cracks. Therefore, when fatigue failure occurs, the debonding failure area of aggregate accounts for a high proportion of the fatigue failure section. Moreover, the lower the stress level, the more significant the bond crack development stage, and the larger the debonding failure area.

## 4. Conclusions

A series of uniaxial fatigue tests was performed to study the fatigue behavior of RAC under compression load. The performance of ITZs in RAC was analyzed using the nanoindentation method. Based on these tests, the effect of ITZs on the fatigue life of RAC was discussed. The conclusions obtained were as follows:

(1) It was found that the fatigue life of RAC is consistent with the two-parameter Weibull distribution, and the *S-N* relationships of RAC under different survival rates were presented.

(2) In the same way as with ordinary concrete, the fatigue deformation of RAC presents a three-stage deformation regularity, and the maximum strain at fatigue failure is in good agreement with the monotonic stress-strain envelope.

(3) Nanoindentation analysis showed that there are three kinds of ITZs in RAC which are ITZ${}_{1}$, ITZ${}_{2}$, and ITZ${}_{3}$. In any kind of ITZ, its indentation modulus is lower than that of the old and new mortars, which are the weak components of RAC. In the high fatigue cycle region with cycle number N $>10^{4}$, the negative effect of ITZs on the fatigue life of RAC is much greater than that of natural aggregate concrete, which is the main reason why the fatigue life of RAC in the high fatigue cycle region is lower than that of natural aggregate concrete.

## Figures and Tables

**Table 1:** Mix proportion of RAC (kg/m${}^{3}$).

Cement	Fly Ash	Sand	Recycled Coarse Aggregate	Water	Additional Water	Water Reducer
288	72	622	1080	143	55	0.648

**Table 2:** Fatigue life of RAC under different stress levels.

Specimen Number	Stress Level	Fatigue Life/*N*	ln*N*	*p*	ln[ln(1/*p*)]
F-0.9-1		17	2.833	0.833	−1.702
F-0.9-2		226	5.421	0.667	−0.903
F-0.9-3	0.9	261	5.565	0.500	−0.367
F-0.9-4		416	6.031	0.333	0.094
F-0.9-5		5329	8.581	0.167	0.583
F-0.85-1		55	4.007	0.833	−1.702
F-0.85-2		137	4.920	0.667	−0.903
F-0.85-3	0.85	3690	8.213	0.500	−0.367
F-0.85-4		5038	8.525	0.333	0.094
F-0.85-5		6879	8.836	0.167	0.583
F-0.8-1		797	6.681	0.833	−1.702
F-0.8-2		2268	7.727	0.667	−0.903
F-0.8-3	0.8	3135	8.050	0.500	−0.367
F-0.8-4		10,982	9.304	0.333	0.094
F-0.8-5		34,139	10.438	0.167	0.583
F-0.75-1		3910	8.271	0.833	−1.702
F-0.75-2		7574	8.932	0.667	−0.903
F-0.75-3	0.75	34,987	10.463	0.500	−0.367
F-0.75-4		91,288	11.422	0.333	0.094
F-0.75-5		380,327	12.849	0.167	0.583

**Table 3:** Weibull distribution parameters and regression analysis results of RAC.

Stress Level	*b*	*c*	R${}^{2}$	*N*a
0.9	0.4092	2.7869	0.8967	907
0.85	0.3668	2.9911	0.8789	3479
0.8	0.5902	5.4416	0.9473	10,096
0.75	0.4661	5.3013	0.9551	87,007

**Table 4:** Fatigue equation of concrete.

Type of Concrete	Fatigue Equation	
	S=1.2144−0.0789logN	$\left(p=0.05,R^{2}=0.8664\right)$
RAC	S=1.0827−0.0736logN	$\left(p=0.05,R^{2}=0.9879\right)$
	S=0.8764−0.0515logN	$\left(p=0.95,R^{2}=0.8590\right)$
NAC-1	S=1.0221−0.05437logN	$\left(p=0.50,R^{2}=0.9914\right)$
NAC-2	S=1.0264−0.05669logN	$\left(p=0.50,R^{2}=0.9999\right)$

**Table 5:** Fatigue life of concrete under various stress levels (*p* = 0.5).

Stress Level	RAC	NAC-1	NAC-2
0.85	1281	1331	1274
0.8	5426	15,240	10,122
0.75	39,632	88,646	73,966

**Table 6:** Mix proportion of NAC (kg/m${}^{3}$).

Concrete Type	Cement	Water	Sand	Coarse Aggregate	Fly ash	Ground Slag
NAC-1	235	180	780	980	90	75
NAC-2	330	175	985	720	60	75

## Data Availability

The data presented in this study are available on request from the corresponding author.
